# Physiological Observations on Double Uterus

**Published:** 1842-12

**Authors:** 


*Physiological Observations on Double Uterus.*—M. Dumas relates in the *Journal de la Soc. de la Méd. Pratique de Montpelier*, a case of double uterus observed by himself, and refers to a number of similar cases, from a comparison of which he draws the following conclusions.

1. The menstrual discharge may continue from the empty uterus, whilst the other contains an embryo.

2. The two uteri do not influence each other to such a degree, as that the empty one should be always under the immediate influence of the other which is fecundated.

3. The woman may be a virgin as regards the left one, and with child in the right, and *vice versa*. She may be at the full time with the one, and in labor, while with the other she is not near her period.

4. Superfœtation may take place in cases of double uterus with double neck and os uteri, and also in cases of double uterus with single neck and os uteri.

5. Notwithstanding the absence of anatomical proof of the existence of superfœtation in cases of double uterus, its possibility ought to be admitted in legal medicine.

6. In well marked cases of double uterus, there will be an inclination to the same side that the fertile womb is on; and all the symptoms will be slightly different from those observed in a case of single uterus.

7. Although it cannot be laid down as a principle, that each cavity of the double uterus can become so developed, as to be able to contain a fœtus at the full period; yet we can believe in frequent abortions.

8. The double uterus, when enlarged, renders labor and the expulsion of the after-birth difficult, and facilitates the laceration of the soft parts.

9. Thanks to the form observed by M. Martin St. Ange, (in which the cavity of the neck of the uterus communicated with that of the body, by a narrow canal thirteen millimetres in length,) we can explain the retention of the secundines, and the difficulty experienced in their extraction, when the canal which retains them has not been sufficiently dilated.—*Amer. Jour. Med. Sci.*, from *Gazette Méd. de Paris*, 4 Feb. 1842.

